# Report of an abscopal effect induced by stereotactic body radiotherapy and nivolumab in a patient with metastatic non-small cell lung cancer

**DOI:** 10.1186/s13014-018-1049-3

**Published:** 2018-05-31

**Authors:** Christian Britschgi, Oliver Riesterer, Irene A. Burger, Matthias Guckenberger, Alessandra Curioni-Fontecedro

**Affiliations:** 1 Department of Hematology and Oncology, University Hospital Zürich, University of Zürich, Zürich, Switzerland; 2 Department of Radiation Oncology, University Hospital Zürich, University of Zürich, Zürich, Switzerland; 3 Department of Nuclear Medicine, University Hospital Zürich, University of Zürich, Zürich, Switzerland

**Keywords:** Anti-PD-1 therapy, Nivolumab, Immune-checkpoint inhibition, Abscopal effect, Stereotactic body radiotherapy, Non-small cell lung cancer

## Abstract

**Background:**

The existence of abscopal effects has been suggested already a long time ago, but only recently with the advent of immune checkpoint inhibition in clinical oncology and modern imaging techniques has it become possible to directly observe such effects in patients. They have been well described in patients with malignant melanoma being treated with immune-checkpoint inhibitors and stereotactic radiotherapy, but experience in other malignancies is very limited.

**Case presentation:**

Here, we describe a case of a patient with metastatic non-small cell lung cancer, who experienced a complete response secondary to an abscopal effect on treatment with anti-PD-1 therapy and stereotactic body radiotherapy to some of the involved sites.

**Conclusions:**

Our case reports confirms the existence of abscopal effects in NSCLC and suggests synergism between immune-checkpoint inhibition and local ablative RT. We suggest that this approach is now further studied in prospective clinical trials on oligo-metastatic or oligo-progressing NSCLC.

## Background

Mole introduced the term ‘abscopal effect’ in 1953 [1]. It describes a phenomenon characterized by tumor regression of untreated metastatic lesions after a local treatment, such as radiotherapy. This is thought to arise because ionizing irradiation causes localized cell death, which induces an immune response called immunogenic cell death. This is triggered by increased antigen release, by improved antigen presentation through increased expression of MHC I on the tumor cell surface, as well as by modulation of cytokines enhancing migration and function of effector CD8+ T cells [2]. However, this event is rare due to immunotolerance at the tumor site, leading to a reduced systemic immune response. Treatment with immune checkpoint inhibitors might overcome tumor-related immunosuppression and start, as well as sustain the immune response towards cancer [3, 4].

## Case presentation

We report on a 47-year-old male current smoker (40 PY), who was diagnosed with lung adenocarcinoma (cT1a pN3 cM0, UICC Stage IIIB). He underwent combination treatment with chemotherapy and cetuximab, followed by radio-therapy in combination with cetuximab and surgical resection as part of a clinical trial (SAKK 16/08; NCT01059188). A pathologically complete response was achieved, but only 8 weeks post-operatively, retroperitoneal lymph node relapse occurred. Since sensitizing mutations were absent, we started palliative chemotherapy (cisplatin / pemetrexed, followed by pemetrexed maintenance). However, maintenance pemetrexed had to be discontinued after two cycles due to severe hematological side effects CTCAE grade 3, requiring in-patient treatment over several days.

**Fig. 1:**
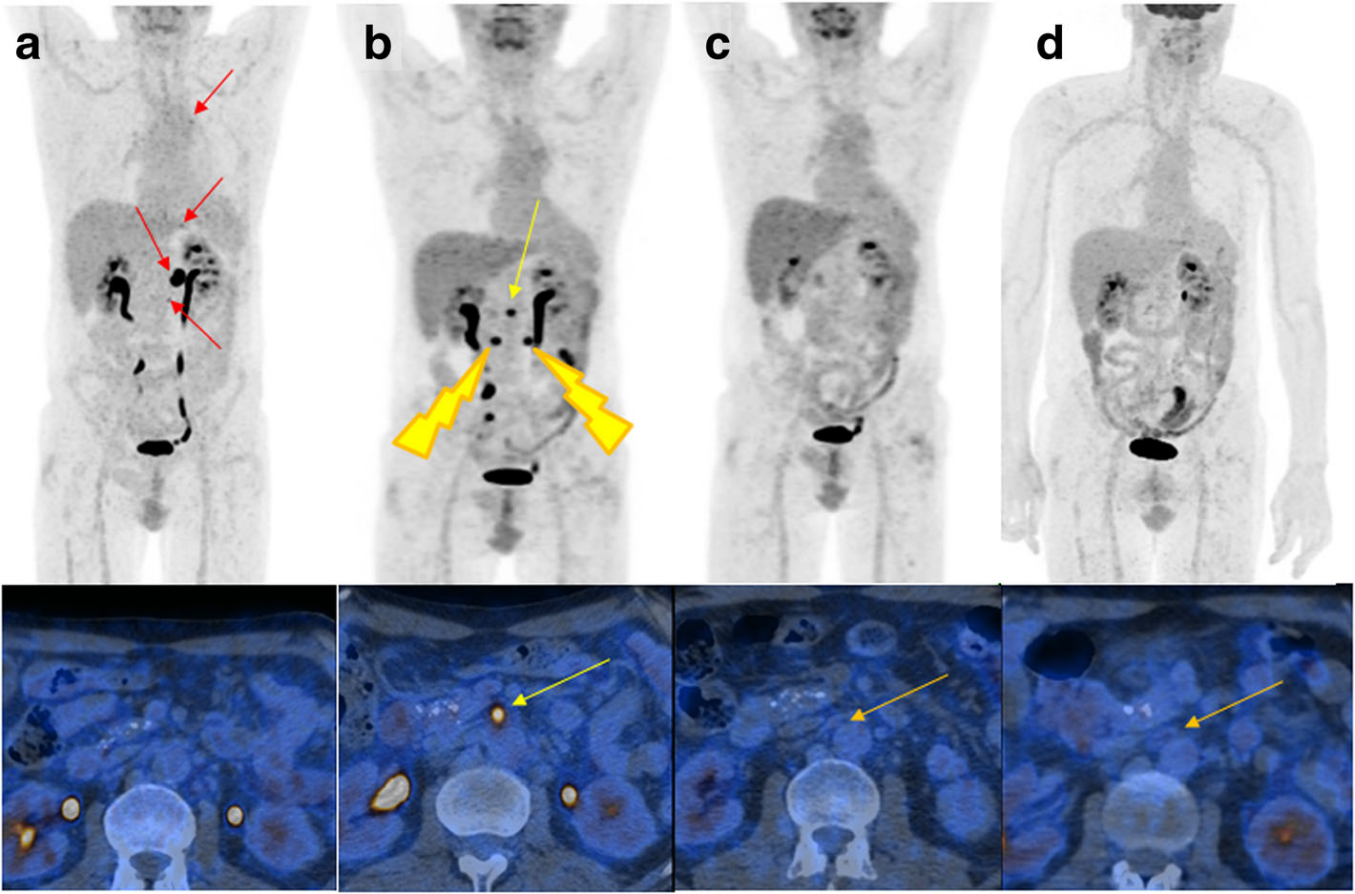
**a** PET/CT staging before start of treatment with nivolumab: the red arrows indicate the localization of the lymph node metastases. **b** PET/CT re-staging after 6 cycles of nivolumab with evidence of complete response of the previous metastases and appearance of new metastases, indicated by yellow arrows. Two out of the three new metastases were irradiated, as indicated. **c** PET/CT restaging 10 weeks after radiotherapy with evidence of complete response. **d** PET/CT restaging two years after start of nivolumab confirming a stable complete remission

After full recovery, a PET/CT scan performed four weeks after hospital demission revealed progressing abdominal lymph nodes (Fig. 1a). The patient was enrolled into an expanded access program (EAP) of the anti-programmed death 1 (PD-1) monoclonal antibody nivolumab. A first PET/CT scan after 6 cycles (i.e. 13 weeks after administration of the first nivolumab dose) showed a mixed response. The initially progressing sites were regressing, but three new abdominal lymph node metastases appeared (Fig. 1b).

**Fig. 2:**
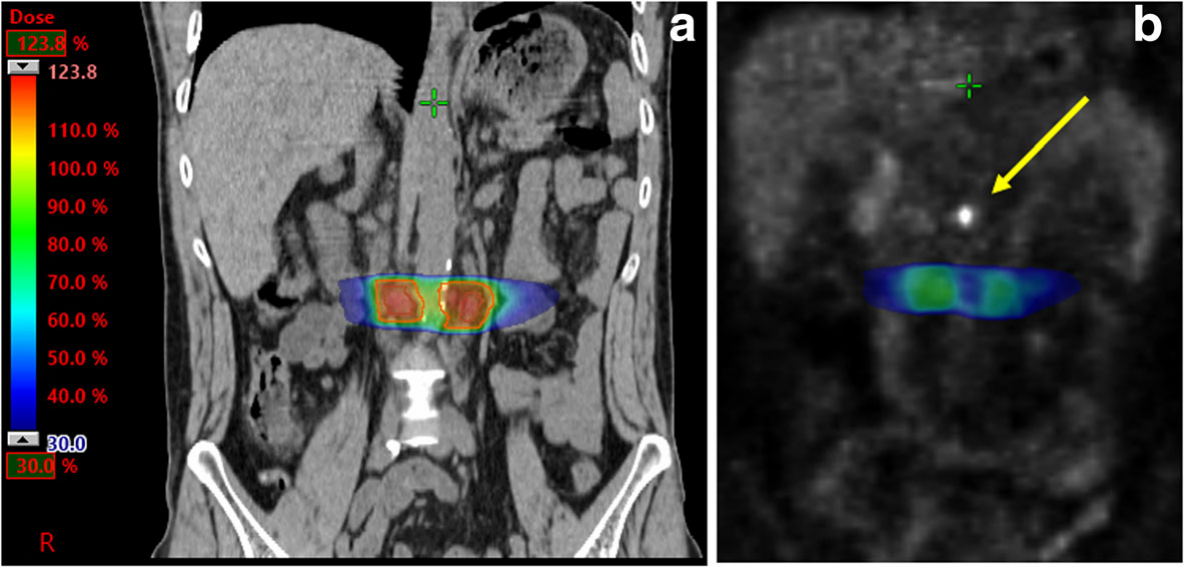
**a** Coronal image of the dose distribution of radiotherapy. The patient received 3 × 6 Gy @ 80%. **b** Image fusion of FDG-PET and treatment plan showing 30% of the prescribed dose (blue) in relation to the untreated FDG-positive lymph node (indicated by the yellow arrow)

The patient was treated with stereotactic body radiotherapy (SBRT) for this oligo-progression (Fig. 2a and b). Two out of the three lymph node metastases were irradiated (3 × 6 Gy = 18 Gy at 80% isodose) (Figs. 1b and 2b). The third lymph node remained un-irradiated because of close proximity to the small bowel and as reference lesion for immunotherapy. It received a radiation scatter dose of 0.4 Gy only, which is far below clinically significant anti-tumor doses. The patient continued treatment with nivolumab during SBRT and thereafter. A PET/CT scan 10 weeks after SBRT (after 13 nivolumab applications in total), showed a complete radiological and metabolic response (CR). Importantly, also the third lymph node metastasis, which had previously progressed and was not irradiated, showed a CR (Fig. 1c). Such a response after initial progression in the absence of any local treatment represents an abscopal effect provoked by PD-1 targeting in combination with SBRT.

During cycle 17, a severe pancreatitis CTCAE Grade 3 occurred and the patient had to be treated for several days as an in-patient, but eventually recovered fully. In the absence of other provoking factors, the most likely differential diagnosis was immune-related pancreatitis and nivolumab was therefore permanently stopped. The patient is today, almost two years after permanently stopping nivolumab, still in complete remission and in regular follow-up in our department (Fig. 1d).

## Discussion and conclusions

Here we describe a case of a patient with metastatic NSCLC experiencing a complete response on treatment with anti-PD-1 therapy and SBRT. A biopsy of the non-irradiated lesion before SBRT was not clinically feasible. Therefore, we cannot principally rule out alternative explanations, such as for example a delayed response to immune checkpoint inhibition. However, the temporal course is highly suggestive of a true abscopal effect.

The existence of abscopal effects has been suggested already several decades ago, but only with the advent of immune-checkpoint inhibitors in clinical routine has it become possible to observe those effects directly in patients. There are several case reports and retrospective analyses suggesting that combining immune-checkpoint inhibition with SBRT might be beneficial in patients with malignant melanoma [5, 6]. In a first report of 101 patients treated with the anti-CTLA4 immune-checkpoint inhibitor ipilimumab, 70 received radiotherapy at some point during their treatment and 31 did not. The median overall survival (OS) in a retrospective analysis was significantly increased in the group, which received RT (19 months vs. 10 months for ipilimumab alone [*p* = 0.01]) [5]. A similar observation was made in a second analysis studying specifically patients who received anti-PD-1 immune-checkpoint inhibition and radiotherapy. Of 59 patients who received pembrolizumab (*n* = 28) or nivolumab (*n* = 31), 17 also received palliative RT. The combination was not associated with increased toxicity and the objective response rate (complete or partial response) was significantly higher in the group, which had received RT (64.7 vs. 33.3%, *P* = 0.02), including one complete responder who exhibited a classical abscopal effect. Such abscopal effects might be especially triggered by RT when limited to the involved region, as showed in preclinical models, in which spearing of draining lymph nodes is crucial to develop antitumor responses [7]. Taken together, these observations indicate that combining RT with immune-checkpoint inhibition (either targeting CTLA4 or PD-1) is well tolerated and has therapeutic potential in malignant melanoma.

Experience in other solid malignancies is more limited, given that immune-checkpoint inhibition first entered clinical routine in melanoma. There are some indications that abscopal effects also exist in NSCLC. A case report described a spontaneous regression of a second pulmonary lesion after having applied SBRT to a first lesion only [8], and there is also a report of an abscopal effect in a patient with adenocarcinoma of the lung receiving a combination of anti-CTLA4 inhibition (using ipilimumab) and SBRT [9]. Moreover, there is evidence from the randomized phase III trial PACIFIC that consolidation immune-checkpoint inhibition using durvalumab after concurrent, definite radio-chemotherapy is beneficial in patients with stage III NSCLC [10].

Our observation in this case now suggests a synergy of concurrent immune-checkpoint inhibition targeting the PD-1/PD-L1 axis and local ablative radiotherapy in NSCLC, as well. This approach should now be studied further in prospective clinical trials in the context of oligo-progressing and oligo-metastatic NSCLC.

## Abbreviations

CR: Complete response
NSCLC: Non-small cell lung cancer
PD-1: Programmed death-1
SBRT: Stereotactic body radiotherapy
